# Supplementary material for: Active site specificity profiling datasets of matrix metalloproteinases (MMPs) 1, 2, 3, 7, 8, 9, 12, 13 and 14
Source: Data Brief. 2016 Feb 22;7:299–310. doi: 10.1016/j.dib.2016.02.036 (PMC4777984; doi:10.1016/j.dib.2016.02.036)
Supplement: Supplementary file 10 — Supplementary material [file mmc10.zip › WebPICS_hMMP13_T_1%/subsite_coop.html]

 

PICS results


- # About
- # Manual
- # Analysis
- # Results

- Redraw
- Seqlogo
- Dependency
- Coop

**Potential subsite cooperativity analysis for hMMP13\_T\_1%**   
Minimum difference for subsite dependency was set to +- 10 percentage-points  
Subsite dependency was checked for all positional occurences above 1 x natural abundance  
**Subsite dependency check is restricted to P3 - P3'**

|  |  |  |  |
| --- | --- | --- | --- |
| Fixed residue | Affected residue(s) | Change (percentage-points) | Vice-Versa change |
| P3\_A | P1\_N | 10.6 | 13.4 |
| P3\_A | P3prime\_N | 10.2 | 24.2 |
| P3\_I | P1\_H | 23.8 | 52.3 |
| P3\_I | P1prime\_C | 12.6 | 17.3 |
| P3\_P | P1prime\_L | -11.1 | -15.0 |
| P3\_V | P1prime\_M | 11.2 | 24.2 |
| P2\_A | P1\_G | 18.8 | 19.6 |
| P2\_A | P1prime\_W | 15.9 | 47.8 |
| P2\_A | P2prime\_K | 12.4 | 24.0 |
| P2\_F | P1\_P | 27.7 | 15.8 |
| P2\_F | P1prime\_V | 38.8 | 19.4 |
| P2\_F | P3prime\_H | 17.3 | 12.6 |
| P2\_F | P3prime\_N | 19.4 | 19.4 |
| P2\_H | P1\_P | 40.2 | 11.5 |
| P2\_H | P1\_W | 23.6 | 47.2 |
| P2\_H | P1prime\_Q | 43.7 | 19.4 |
| P2\_H | P2prime\_Q | 41.6 | 13.9 |
| P2\_H | P3prime\_H | 42.3 | 15.4 |
| P2\_M | P2prime\_M | 47.2 | 47.2 |
| P2\_Q | P1prime\_M | 20.8 | 27.7 |
| P2\_Q | P3prime\_S | 28.4 | 17.5 |
| P2\_S | P1\_S | 22.5 | 32.9 |
| P2\_S | P2prime\_V | 11.6 | 10.5 |
| P2\_V | P3prime\_A | 25.9 | 11.6 |
| P1\_A | P1prime\_I | 13.1 | 15.4 |
| P1\_A | P3prime\_A | 19.7 | 13.6 |
| P1\_D | P1prime\_C | 12.6 | 17.3 |
| P1\_D | P2prime\_H | 12.6 | 17.3 |
| P1\_D | P3prime\_T | 11.9 | 14.5 |
| P1\_G | P1prime\_W | 16.8 | 48.5 |
| P1\_G | P2prime\_K | 17.1 | 31.8 |
| P1\_G | P3prime\_A | -12.6 | -11.3 |
| P1\_G | P3prime\_G | 12.6 | 12.6 |
| P1\_H | P2prime\_Q | 31.6 | 13.2 |
| P1\_N | P1prime\_H | 10.5 | 39.5 |
| P1\_N | P2prime\_V | 25.3 | 18.1 |
| P1\_P | P1prime\_V | 17.4 | 15.2 |
| P1\_P | P2prime\_Q | 20.2 | 23.5 |
| P1\_P | P2prime\_T | 15.8 | 27.7 |
| P1\_P | P3prime\_H | 28.0 | 35.7 |
| P1\_Q | P1prime\_M | 12.5 | 12.5 |
| P1\_Q | P2prime\_I | 55.5 | 20.8 |
| P1\_Q | P3prime\_T | 43.7 | 29.1 |
| P1\_S | P2prime\_L | 13.3 | 12.3 |
| P1prime\_C | P2prime\_T | 19.4 | 19.4 |
| P1prime\_C | P3prime\_T | 18.7 | 16.6 |
| P1prime\_I | P3prime\_D | 13.7 | 16.7 |
| P1prime\_M | P2prime\_W | 30.5 | 45.8 |
| P1prime\_M | P3prime\_S | 24.2 | 11.2 |
| P1prime\_Q | P2prime\_Q | 36.0 | 27.0 |
| P1prime\_Q | P3prime\_H | 14.5 | 11.9 |
| P1prime\_V | P2prime\_I | 13.8 | 13.8 |
| P1prime\_W | P2prime\_K | 45.8 | 29.4 |
| P2prime\_I | P3prime\_N | 19.4 | 38.8 |
| P2prime\_K | P3prime\_D | 11.6 | 11.6 |
| P2prime\_L | P3prime\_A | 22.6 | 10.9 |
| P2prime\_Q | P3prime\_H | 25.6 | 28.0 |
| P2prime\_T | P3prime\_S | 40.9 | 25.2 |
| P2prime\_W | P3prime\_S | 40.9 | 12.6 |

  
**PICS analysis of protease: hMMP13\_T\_1%**  
143 cleavage sites analyzed  
PICS library made with (T)rypsin, (G)luC or (C)hymotrypsin:   
Cutoff for graphic display: 2 x natural abundance  

|  |  |
| --- | --- |
| Positional occurences  (table for total and relative (in %) values) | Occurences relative to natural abundance (table) |
|  |  |
